# Error management for musicians: an interdisciplinary conceptual framework

**DOI:** 10.3389/fpsyg.2014.00777

**Published:** 2014-07-25

**Authors:** Silke Kruse-Weber, Richard Parncutt

**Affiliations:** ^1^Department of Music Education, University of Music and Performing ArtsGraz, Austria; ^2^Centre for Systematic Musicology, University of GrazGraz, Austria

**Keywords:** error friendliness, error management, risk management, risk-taking, error prevention, music performance, instrumental music pedagogy, metacognition

## Abstract

Musicians tend to strive for flawless performance and perfection, avoiding errors at all costs. Dealing with errors while practicing or performing is often frustrating and can lead to anger and despair, which can explain musicians’ generally negative attitude toward errors and the tendency to aim for flawless learning in instrumental music education. But even the best performances are rarely error-free, and research in general pedagogy and psychology has shown that errors provide useful information for the learning process. Research in instrumental pedagogy is still neglecting error issues; the benefits of risk management (before the error) and error management (during and after the error) are still underestimated. It follows that dealing with errors is a key aspect of music practice at home, teaching, and performance in public. And yet, to be innovative, or to make their performance extraordinary, musicians need to risk errors. Currently, most music students only acquire the ability to manage errors implicitly – or not at all. A more constructive, creative, and differentiated culture of errors would balance error tolerance and risk-taking against error prevention in ways that enhance music practice and music performance. The teaching environment should lay the foundation for the development of such an approach. In this contribution, we survey recent research in aviation, medicine, economics, psychology, and interdisciplinary decision theory that has demonstrated that specific error-management training can promote metacognitive skills that lead to better adaptive transfer and better performance skills. We summarize how this research can be applied to music, and survey-relevant research that is specifically tailored to the needs of musicians, including generic guidelines for risk and error management in music teaching and performance. On this basis, we develop a conceptual framework for risk management that can provide orientation for further music education and musicians at all levels.

## INTRODUCTION

A century ago, sound recording technology transformed performance. The main source of classical music became error-free studio recordings by great artists. Audiences became accustomed to perfection. Music students correspondingly strived for immaculate performances, becoming less adventurous and individual. Exaggeration of the importance of errors at the expense of other aspects of musicianship affected university admission procedures, competitions, instrumental teaching, and learning. The classical music world increasingly identified with a perfectionist, error-free esthetic that contrasted with, and increasingly diverged from, the more unconventional improvisatory esthetic of jazz (Hamilton, 2003). In the words of Martin (1996–2007), “Jazz is an art form that must strive for greatness on all levels; (…) if the artists are not encouraged to take risks, how can greatness ever be achieved? There are no sure things in life or art.”

Musicians at all levels of proficiency across their lifespan must deal with errors and develop strategies that balance the vitality of risk-taking against error prevention in both practice and performance (Westney, 2003). In almost every learning process, miscues occur because of insufficient knowledge and skills, or as a consequence of inappropriate goals and or inadequate planning (Zapf et al., 1999). If mistakes are approached in a negative or destructive way, the fear of errors can induce state anxiety and stage fright, which in turn affects performance quality (Möller, 2004). But error-based training can produce more adaptive training transfer than error-avoidance drills (Heimbeck et al., 2003; Keith and Frese, 2005).

Live music performances are rarely perfect, and imperfections often cause considerable chagrin. But performers and audiences often perceive errors quite differently. In general, the perception of errors is context-, listener-, and situation-dependent; blunders that seem catastrophic to musicians on stage may go unnoticed by the audience (Repp, 1996). The audience may not realize some tones are cut short (not held for their notated duration) or if their pitch is changed (substitution error) such that the new tone still fits the harmonic context (Groulx, 2013). Repp (1996) also pointed out that some kinds of perceptually insignificant pitch errors, such as tendencies to fill chords, could be viewed as positive: they are spontaneous liberties that may enhance the esthetic effect of music. Nearly all errors in piano performances occur in non-melody voices, often inside chords. Flossmann et al. (2011) investigated faults in performances on a computer-controlled Bösendorfer piano by Nikita Magaloff in 1989.

Flossmann and Widmer (2011) classified errors that occur in different contexts and patterns. Forward- and backward-related errors that may be attributed to memorization problems, repeated notes that are possibly caused by changes of fingering, non-harmonic errors that occur mostly in octave runs, insertions or substitutions that do not disrupt the harmonic context, tied notes that are played again, or successive notes of the same pitch that are played only once to reduce technical difficulty. Systematic inaccuracies occur in more than 60% of instances of the same or an analogous context and most can be attributed to either technical or memory problems (e.g., omitted inner voices). Note order slipups are related to timing and occur more often at fast tempos in descending patterns with two or more successive notes or a downward sequence of several notes suggesting intentional simplifications. According to Palmer and van de Sande (1993), pitch errors can be coded on dimensions such as size (single note, chord, or note–chord combination), source (contextual/non-contextual), type (addition, deletion, substitution, shift), and movement (anticipatory/perseveratory; Palmer and van de Sande, 1993).

Palmer and van de Sande (1993) and Gudmundsdottir (2010) investigated cognitive aspects of errors in children’s piano performance, and confirmed Repp’s (1996) earlier finding that melodic voices contain fewer errors. On this basis, they considered conceptual, retrieval-based, and articulatory influences on knowledge that forms cognitive plans for music performance. Beginners’ errors are often obvious and easily detected. As skill levels increase, errors become less frequent and more subtle (Repp, 1996; Gudmundsdottir, 2010). Children with more musical training show quicker detection and correction of faults more anticipatory and less perseveration behavior, and a longer range of planning than children with less training (Palmer and Drake, 1997). Gudmundsdottir (2010, p. 65) identified three basic pitch-error types in the children’s music reading performances: “Erroneous pitches (pitches that did not match the target pitches in the score), redundant pitches (correct pitches that were repeated as in hesitation) and omitted pitches.” Beginners are also more likely to repeat errors, either because they do not realize there was an error, or because they are unable to correct it in repeat performances (Barry and Hallam, 2002).

Most importantly for our approach, Zapf et al. (1999), Flossmann et al. (2011), and Maidhof (2013) found that expertise does not generally lead to performance perfection. Instead, highly trained musicians manage and correct errors faster and more easily: one learns to create an impression of accuracy in a performance that is actually far from faithful to the score (Sloboda, 1985; cited in Repp, 1996). In other words, experts manage errors better, but it is unclear whether this process is conscious or implicit. One might expect the best performer-teachers to pass on skills of error management to their students. We are instead confronted with a striking lack of explicit or theoretical knowledge about error management and learning from blunders in music performance.

The skill of managing errors quickly and easily can also be described as error management. Miles Davis explained, “If you hit a wrong note, it’s the next note that you play that determines if it’s good or bad” (Faust and Marsalis, 2013). In a similar vein, Barenboim (2012) commented that “The art of performance is not to not play out of tune. The art is to play out of tune and yet make it sound right.”

This study aims to lay the foundations for a new theory of error management in music practice and performance that draws upon research in a series of relevant disciplines. The structure of this review paper is, first, to focus on concepts of error management in education, psychology (work, organizational), economics, and sports, as well as high-risk disciplines such as aviation and medicine. Second, we look in detail at three approaches to error management: *risk management* (which aims to prevent errors in performance), *error management* (which attempts to minimize the consequences of errors when they occur), and *error-friendly teaching and learning* (a metacognitive approach to error tolerance in musical practice). Finally, we will describe the possible implications for musicians applying the principles and concepts of risk and error management to the musical environment.

Although error and risk management are important issues in many disciplines, and although errors occur frequently in learning processes, little is known in instrumental pedagogy about systematic strategies for managing errors. The purpose of the present interdisciplinary approach is to apply ideas, principles, empirical results, and practical strategies in the area of risk and error management to the training of musicians.

## THEORETICAL BACKGROUND

### GENERAL PEDAGOGY

For years, many educators promoted the idea of errorless learning. The challenge confronting students was to avoid errors altogether. Skills were acquired by repetition without reflection. The idea embedded in this approach is that if students make errors, they will internalize them and be prevented (or slowed) in learning the correct information (Roediger and Finn, 2009).

Empirical research in general pedagogy (e.g., Oser and Spychiger, 2005) has suggested that the productive and creative potential of errors is not used to full capacity. In educational contexts, errors may be accepted as unavoidable incidents, but generally they are not considered to be helpful, so they are not even categorized in educational discourse (Weingardt, 2004). In learning situations, where they could be informative and provide positive learning opportunities, students may merely avoid them.

As educational theory has increasingly been influenced by constructivist ideas, the focus has shifted to a detailed consideration of the learning process. In a constructivist perspective, errors are useful and positive sources of information for further learning (Spychiger, 2006). Exploration respectively varied practice and playing provides individuals with unstructured opportunities to explore effective strategies, in which self-directed learning is encouraged. The contrast between behaviorist and reflective approaches to teaching is also found in approaches toward dealing with errors (Kruse-Weber, 2012a). In the behaviorist approach, a teacher will judge a student’s performance unilaterally, without giving advice on how to correct flaws. In this approach, teachers are like police, monitoring and controlling the performance of their students but not helping them to learn. Teachers who are interested in the cognitive and emotional processes that lie between input (score) and output (performance) should avoid the temptation to immediately correct errors. Instead, they regard student errors as opportunities for creative pedagogical intervention. They should adopt a constructivist attitude that acknowledges the active role of the student in generating her or his own knowledge in interaction with the teacher, and the important role of metacognition in training students to learn more independently to ensure their long-term success. They should ask simple, process-oriented, metacognitive questions such as: why did you play this? what did you think about it? which aspects were important for you? what aspect are you focusing your attention on? where are you looking? Questions of this kind encourage the student to think about what he or she is doing (Kruse-Weber, 2012a).

### PSYCHOLOGY

In behavioral psychology (Skinner, 1953), errors were equated with punishment that can inhibit behavior but do not contribute to learning. Skinner regarded errors as a consequence of moving too fast from one step to the next in a learning program, or a lack of the prerequisite behaviors that are necessary for learning to succeed. On this basis, if we are to prevent learners from making errors, we must offer them detailed, step-by-step instructions for the correct performance of specific tasks and the correct solution of specific problems.

In his social-cognitive theory, Bandura (1977) viewed errors as needless, time consuming, and hence detrimental to learning. Instead, he favored the idea of a guided and error-free learning environment. According to (Bandura, 1986, p. 47; in Keith and Frese, 2008, p. 60), learners should be “spared the costs and pain of faulty effort.” Students should instead receive guidance that leads to flawless behavior.

Traditional approaches to teaching involve a kind of linear thinking, based on a linear understanding of causality. Assuming that the same cause generally leads to the same effect, the teacher focuses entirely on the result of the performance – the outcome behavior – rather than considering what might be happening inside the “black box” of the student’s psychology and physiology. Learners are thus guided by a step-by-step series of strategies. Teachers often wait for one correct answer to a question; they fail to realize that there may be many possible solutions, and that errors have productive potential. In a cybernetic approach, Von Foerster and Pörksen (2008) commented that linear systems of teaching “dumb children down” to “trivial machines” (Von Foerster and Pörksen, 2008, pp. 54–55).

In a more constructivist approach, Bruner (1960, 1961) regarded learning as an active process associated with problem solving. Learners are constantly constructing new ideas or concepts based upon interactions between their experience and their existing knowledge. The learning process involves metacognitive skills of selection and transformation of information, decision making, hypothesis generation and testing, and meaning generation based on available information and experiences. An active process of discovery allows the student to uncover the interrelationships between concepts and ideas, which in turn allow them to gain new knowledge. Making errors is a necessary aspect and gives the learner a better comprehension of the information. Learning and knowledge evolve through active interaction with one’s environment through trial-and-error experimentation. Constructivism is about how learners devise their own meaning by asking questions, developing answers and interacting and interpreting the environment. Wills (2007) confirmed this approach of learning; he demonstrated that we learn more rapidly about cues for which we initially make incorrect predictions than cues for which our initial predictions are correct.

Traditional models of learning have recently been questioned because of their linear approach. Learners typically start with the same exercise followed by other equal teaching exercises. While learners in traditional pedagogical principles need to progress “from easy to hard” or “from simple to complex,” the differential approach is based on a non-linear understanding of causality, where small causes can lead to big effects and vice versa. The differential learning approach, which is common in sport, tries to find individual optimal performance patterns by way of a large variety of between-exercises differences by the systematic avoidance of repetition and constantly changing movement tasks which add stochastic perturbations, based on the principle of balancing solutions in a certain range of solutions. Research from Schöllhorn et al. (2012) has provided evidence for the superiority of a differential learning approach for mastering single movement techniques, in comparison to repetition- and correction-oriented approaches.

Results of error studies by Keith and Frese (2008) in the area of work and organizational psychology have been consistent with the negative connotations of errors in educational research. Working situations are often similar to learning situations in that errors are seen as a nuisance. They interrupt the workflow; error correction is time consuming and causes frustration, which in turn causes stress; and some errors have severe consequences for individuals and organizations that can lead to desperation. It is therefore understandable that people usually prefer to avoid errors in the first place. But research in software design (Keith and Frese, 2008) found that error management training (EMT), in which students are free to make errors, ultimately leads to higher adaptive transfer when compared with error-avoidance training because errors during training stimulate attention, which in turn facilitates later retrieval of similar problems and their solutions (see also Zapf et al., 1999; Badke-Schaub et al., 2008; Spitzer, 2008; Kornell et al., 2009).

The theoretical foundation of EMT is action theory (Frese and Zapf, 1994; Hacker, 1998). Action theory views errors during training as valuable pieces of information, because they serve as feedback for one’s actions and can point out what aspects of one’s knowledge need further correction and refinement. In early studies (Frese, 1991, 1995; Frese and Zapf, 1991; Frese et al., 1991), EMT was applied to the teaching of software skills. Other studies investigated decision-making tasks (e.g., Gully et al., 2002) or applied EMT to driver training (Ivancic and Hesketh, 2000). We will describe further issues of EMT in the section “Error Management Training in Human–Computer Interaction Studies (EMT).”

### INSTRUMENTAL MUSIC PEDAGOGY

In the late 1950s and early 1960s, cognitive psychology displaced behaviorism as the leading paradigm in both psychology and educational research. Psychological interest shifted away from stimulus-response relationships to the processes that lie between stimulus and response (Hoffmann, 2003). But this paradigm shift had little impact on music instrumental pedagogy. Musicians still have a generally negative attitude toward performance errors, which are equated with failure, shame, and fear. Related to this, learning and performing situations are not sufficiently differentiated, and support and evaluation procedures are not transparent enough (Spychiger, 2012). In instrumental music education, there is a tendency to focus on unilateral error prevention rather than learning from errors.

We have contradictory situations in instrumental pedagogy. On the one hand, beginners leave errors uncorrected because they do not have schemata in place for correcting them in practice (Hallam, 2001; Hallam et al., 2012). On the other hand, there is a strong focus on error prevention in teaching and learning. An error-friendly culture of learning that cultivates an optimistic, enlightened attitude toward errors could resolve this contradiction. Error friendliness is a way of offering students more opportunities to learn by reducing the negative impact of undesired outcomes (Spychiger, 2012). Music psychologists have confirmed that a constructive approach to errors incorporates informative feedback and error correction during the learning progress (Ericsson et al., 1993; Ericsson, 1996).

There seem to be another contradictory situation in music: While some musicians strive for risk-taking to achieve greatness in the performance, for many others excellence means flawless performance and most possible security. They go to extremes in practicing perfection in a desperate attempt to avoid risks. We have always to balance risk taking vs risk avoidance.

The philosopher of jazz (and music), Marsalis (2013) has argued that learning arts or music means to constantly demand risks. Art education gives students skills to create, adapt, and take risks in the future and therefore dealing with one’s inevitable mistakes is part of an artist’s education. Coleman Hawkins, the American jazz saxophonist, once said, “If you don’t make mistakes, you aren’t really trying.” Risk-taking is about experimentation and pushing boundaries in ways which musicians themselves may not be sure will work. It demands courage, curiosity, and desire, and a degree of spontaneity. Successful risk-taking should be shaped by skill and instinct and be managed, but not avoided. The biggest risk may be to take no risks at all (McMaster, 2008). Miles Davis, for example, was a “fanatic for interplay among musicians – sometimes even assembling bands with a trouble maker that would challenge the other players. His vision of music demanded spontaneity where each musician was in the moment” (Garnett, 2013).

## THREAT, RISK, AND ERROR MANAGEMENT IN HIGHER RISK DISCIPLINES AND ORGANIZATIONAL DOMAINS

### DEFINITIONS

To clarify the discussion, it is useful to consider definitions of central terms. The term “risk” is traditionally negative: it is the “agent of damages, dangers and negative effects” (Mitschele, 2009; p. 10; see also Langeroodi et al., 2011). “Threat” is similarly negative, but people who are “threatened” are more passive than those who take “risks,” and the consequences of a “threat” may also be more negative. Defined as incidents that occur outside the flight-deck, threats are conditions that have the potential to impact negatively the safety of a flight (Thomas, 2004). The term “risk” allows for the possibility of positive outcomes. In addition, the cognitive appraisal of risks is subjective, influenced by individual perception.

More generally, risk is the “effect of uncertainty on objectives”; in this context, an “effect” may be either a positive or a negative deviation from what is expected (see ISO 31000, International Organization for Standardization, 2009). Whenever trying to achieve an objective, there is always the chance that things will not go according to plan and the results are not as expected. Results are sometimes positive, sometimes negative, and sometimes both. One aim of ISO 31000 is to reduce uncertainty as much as possible. “Uncertainty (or lack of certainty) is a state or condition that involves a deficiency of information and leads to inadequate or incomplete knowledge or understanding. In the context of risk management, uncertainty exists whenever your knowledge or understanding of an event, consequence, or likelihood is inadequate or incomplete*”* (Praxiom Research Group, 2010–2013). Distress is triggered not only by the dynamics of information processing but also by the subjective assessment of the individual situation. An excess of information is objectively perceived as overload and subjectively as a threat. This is reflected in thoughts such as “I do not manage this” or “I cannot stand it anymore” (Manz, 2006, p. 41).

The error is defined as the unintended result of an action (see e.g., Maidhof, 2013). Error management occurs during and after an error. The main aim is not to avoid the error itself, but to avoid its negative consequences, and to resolve errors easily, quickly, and without stress (Zapf et al., 1999). To achieve this goal, it helps to develop a flexible and emotionally relaxed attitude toward errors. Error management involves understanding the nature and extent of the error and identifying behavioral responses that can prevent errors or mitigate their effects (Helmreich, 2000). Disturbances are dealt with in a differentiated manner that does not significantly compromise the initial goals (Weingardt, 2004).

Maher (2012) lists the key aspects of the “failure tolerance chain” (p. 201):

(1) Taking risks produces a mixture of failures and innovation.
(2) Failure tolerance can be a step toward innovation.
(3) Innovation is often successful, therefore:
(4) Failure tolerance can lead to success

Learners are faced with a paradox. On the one hand, they tend to fail when they are trying to do something innovative. On the other hand, if they do not fail, they are not innovative. Risk and error management techniques are systematic attempts to resolve this paradox.

### GENERAL APPROACHES

The conceptual framework of error management has been used in different complex organizational domains, including higher risk disciplines such as aviation, industry, and medicine, to develop a better understanding of the processes of error systems and on that basis to develop better strategies to manage errors (Helmreich, 1998). This is as true in industrial and organizational psychology, where the terms “risk management” and “error management” are usual, as in aviation, where the concept of “threat and error management” (TEM) is common (Helmreich et al., 1999). In normal flight operations, flight crews are faced with a variety of external threats and can commit a range of errors that could affect the safety of airline operations. TEM is used to evaluate both the performance of individual pilots and the environment in which they work (Koglbauer, 2009). It occurs prior to a potential error and involves anticipating errors and associated disruptions. This proactive approach is an attempt to detect and evaluate errors, and to mitigate risk factors and facilitate the avoidance of incidents and accidents (Langeroodi et al., 2011).

The medical profession could learn from TEM in aviation. Surveys have confirmed that pilots and doctors have common interpersonal problem areas and similarities in professional culture (Helmreich, 2000). In aviation, accidents are usually highly visible; as a result, aviation has developed standardized methods of investigating, documenting, and disseminating errors, and lessons drawn from them (Helmreich, 2000). Aviation increasingly applies procedures of error management; the systematic observation of flights in operation has identified failures of compliance, communication, procedures, proficiency, and decision making in contributing to errors (Helmreich, 2000).

Psychologists in the area of human factors acknowledge that errors do not occur in isolation, but are generally the result of failures at a systemic level (Reason, 1997). In general, an error committed by a pilot cannot be clearly separated from the complex situation in which the pilot is working. Helmreich points out that accepting the inevitability of errors and the importance of reliable data on error and its management will allow systematic efforts to reduce the frequency and severity of adverse events. Error management adopts a non-punitive stance toward inadvertent error, which promotes an error-friendly culture. It involves error tolerance, which in turn involves being prepared for disruptive events and adverse developments (Helmreich, 1998). Certainty is not merely about the absence of errors; it involves managing errors and disturbances to reduce the probability of losses while still achieving given objectives (Thomeczek and Ollenschläger, 2004).

There is an interaction between safety and human performance. Performance may fall below expectations because risks were discovered too late, or simply ignored. In this regard, mere knowledge of risks and possible responses to error is not enough; it is also necessary to practice their application in natural situations. Trainee pilots experience critical situations in flight simulators. They are familiar at this level with the consequences of errors such as poor decisions or incorrect responses. They internalize reactions to errors that may happen in a future emergency (Oser et al., 1999). But there is the risk of a *Rumpelstiltskin effect*: although these pilots may be able to demonstrate their practical knowledge in a flight simulator, they may still react inappropriately when unexpected incidents or errors occur during a real flight. The solution is to practice dangerous stunts with no passengers – to develop behavioral-strategies responding to threats under stressful conditions (Kruse-Weber, 2012b).

In the TEM concept, principles of anticipatory learning theory are applied to flight instruction practice. The concepts of anticipatory processes assume that our behavior in complex situations is not only a reaction to situational conditions; behavior is also affected by expected outcomes (Kallus et al., 1997; Maidhof et al., 2009); “We do not respond to what we see, but we see what we expect” (Kallus, 2009, p. 9). The findings of Kallus and Tropper (2007) provide evidence for the existence of anticipatory, feed-forward control mechanisms involved in the regulation of resource allocation during high task load (Kallus and Tropper, 2007; in Koglbauer, 2009).

Distress before an accident or triggered by the first few seconds of a crisis might reduce the quality of information processing and error management performance (Wickens, 1992; in Koglbauer, 2009). Although flight training in simulators is used all over the world, only a few studies have evaluated the transfer of skills acquired in the simulator to real flight (Koglbauer, 2009). Situation awareness in a complex environment is the result of “perception, comprehension and projection of goal relevant information” (Koglbauer, 2009, p. 10). These empirical findings have important practical consequences: anticipation-based TEM in flight simulators can improve the TEM performance of pilots, while decreasing workload and emotional strain. Koglbauer (2009) noted that as a consequence of anticipation-based recovery training in the simulator, pilots in an experimental group demonstrated a significant increase in performance, as well as a decrease in workload and emotional strain during both a simulator test and the flight test performed in the aircraft. Pilots’ good mood and positive emotions (i.e., strength, pride, enthusiasm, and excitement) improved significantly as a consequence of repeated flight sessions. “Pilots of the control group who did not receive specific recovery training in the simulator seemed to learn during tests and improved their performance significantly from the first flight session, but their performance was clearly inferior to the experimental group” (Koglbauer, 2009, p. 143).

### ERROR MANAGEMENT TRAINING (EMT) IN HUMAN–COMPUTER INTERACTION STUDIES

As pointed out above, recent studies in the psychology of human–computer interaction have shown that specific EMT has even more positive effects when it is supported by additional metacognitive advice. In studies on the psychology of human–computer interaction by Keith and Frese (2005), participants received brief instructions that emphasized the positive informational feedback of errors. They were told that it is natural to make errors. Some examples should illustrate these encouraging brief instructions in EMT (after Keith and Frese, 2008):

• The more errors you make, the more you learn!
• You have made an error? Great! Because now you can learn something new!
• Errors are a natural part of the learning process!
• There is always a way out of an error situation!
• Errors inform you about what you can still learn!
• See the potential of your errors!
• Everybody makes errors, so don’t worry about them, you will learn from them!

The researchers demonstrated that EMT encourages emotional control and metacognition (in short, you learn to learn or “thinking about thinking”, Barry and Hallam, 2002, p. 154). Students reflect on the causes of errors and develop an emotionally relaxed attitude toward them, which improves learning. Metacognitive activities prompt learners to stop and think about the causes of the error and to experiment with different solutions. Two major verbal categories can be distinguished: metacognitive (e.g., “if I do this here, I should be able to do that there”) and task-oriented statements (e.g., “now I do this, then this…that’s because I did this…”; Keith and Frese, 2005, pp. 681–682). Learners can develop two different self-regulatory skills: they learn to exert emotional control to reduce negative emotional reactions to errors and setbacks (Kanfer et al., 1996; in Keith and Frese, 2008), and they can engage in metacognitive activities that involve planning, monitoring, and evaluating one’s progress during task completion and revision of strategies (Brown et al., 1983).

Error management training goes beyond regarding errors as negative feedback indicating non-achievement of a goal. Rather, learners are encouraged to use errors as a basis for thinking ahead and trying out something new. The focus on informative aspects of errors is a distinctive feature of the error management approach. Error management training involves active exploration as well as explicit encouragement for learners to make errors during training and to learn from them. Participants learn to reduce negative emotional reactions to errors and setbacks; they then engage in metacognitive activities that involve planning, monitoring, and evaluating” (Keith and Frese, 2008). Error management training is distinguished from alternative training methods as purely exploratory and proceduralized training. In active exploration, participants are given minimal guidance and they actively explore and experiment on their own; adequate mental models are best acquired by direct action. In proceduralized training, participants are given a clear feedback and they are not encouraged by errors during training. Another distinguishing feature is that EMT creates a learning environment in which errors are likely to occur. It creates a positive error climate, but there is still a residual fear of errors, which of course must be corrected (Spychiger and Oser, 2005).

A meta-analytical study by Keith and Frese (2008) across 24 empirical studies (*N* = 2,183) revealed that EMT is more effective in tasks for which there is clear feedback. Early studies reported EMT to be effective in terms of post training transfer than for within-training (Keith and Frese, 2008).

### CLASSIFYING AND PROCESSING RISKS

Risks can generally be perceived as either failures or opportunities. Balancing between both aspects means changing conditions or removing sources of risk. In order to identify risks and figure out how best to mitigate them, the entrepreneur Hirai (2010) provides a conceptual framework for classifying risks in the dimensions likelihood of occurrence, severity of the potential consequences and reversibility (see also the classification of consequences and reversibility on errors from Spychiger in Kruse-Weber, 2012c; p. 32). Low consequences and high reversibility consideration and a relatively low likelihood of occurring how people face up to risks – he calls them “ignorable risks” – do not need spending a lot of time worrying about. Then he gives the category of “nuisance risks” – little things that often seem to go wrong – whose impacts are easy enough to be minimized or mitigated through straightforward simple changes in behavior. Some examples for this category are demonstrated below (see A Conceptual Risk Management Framework for Musicians). There are countless examples of “nuisance risks” and their simple solutions. Risks that could have major consequences but are relatively unlikely to happen are often “insurable risks.” The risks with high likelihood and high consequences involve the “risks to actively detect, monitor and mitigate” (Hirai, 2010).

ISO 31000 is a family of standards relating to risk management codified by the International Organization for Standardization. The purpose of ISO 31000:2009 is to provide principles and generic guidelines, assessment techniques, and definitions on risk management. ISO 31000 seeks to provide a universally recognized paradigm for practitioners and companies employing risk management processes. The following responses to risk are considered in the ISO guidelines:

(1) Avoiding the risk by deciding not to start or continue with the activity that gives rise to the risk.
(2) Accepting or increasing the risk in order to pursue an opportunity.
(3) Removing the risk source.
(4) Changing the likelihood.
(5) Changing the consequences.
(6) Sharing the risk with another party or parties (including contracts and risk financing).
(7) Retaining the risk by informed decision

These guidelines clearly demonstrate that risk-taking can be seen as a deliberate strategy. With this checklist the person is able to analyze, evaluate, balance, and decide which aspect is the best for further action in the decision-making processes. Risk identification is a process itself. It involves “finding, recognizing, and describing the risks” that could affect the achievement of an organization’s objectives. Additionally, the person can add informed opinions, expert advice, and stakeholder input to identify the organization’s risks (Praxiom Research Group, 2010–2013).

After having identified the potential risks or vulnerability of critical effects, one can list and analyze risks. Analysis includes the evaluation of the scenarios according to the criteria of probability and consequence potential. It also includes the analysis of causes or failures. The next step is to plan possible responses to the risk and to identify ways to reduce negative outcomes. Through simulation and anticipation of risk situations in practice, one can monitor whether new skills are implemented correctly. At least the person has to prioritize the risk reduction based on a certain strategy.

The flip side of risk is opportunity. Risk-taking can lead to success. The person allows and analyzes failures with the objective of improving the quality of subsequent risk-taking. This risk-taking is not only intelligent – it can produce interesting results. The following well-known quotation from playwright Edward Albee resonates with this notion “If you are willing to fail interestingly, you tend to succeed interestingly” (Albee; in Maher, 2012, p. 202). Maher argues that entrepreneurs, if they want to succeed, must take significant risks. Entrepreneurship is neither easy nor risk-free. He points out that more than half of all startups fail within a few years. Risk is an integral part of entrepreneurship. Great entrepreneurs and great artists achieve success through keen awareness and management of risks (Maher, 2012).

### SUMMARY

**Table 1** illustrates three approaches to error management, and summarizes the most important objectives, issues, methods, and motives. All three approaches can be seen as error-friendly: errors are looked at and analyzed as positive sources of information rather than ignored.

**Table 1:** Approaches to error management strategies.

	Risk and threat management	Error management training (EMT)	Error management
When	*Before the error*	*After the error*	*During and after the error*
Why	An “early warning system” through threat recognition and the implementation of error avoidance behavior	Generating emotionally stress-free attitude toward errors through understanding, managing and preventing errors	Using the creative potential of errors managing them constructively and fast
How	Asking *What could go wrong?* Anticipating risks, threats and disturbances through simulation during practice. Having realistic expectations	Developing metacognitive and task-orientated strategies	Minimizing negative consequences of errors through constructive cognitive appraisal or fast error corrections
Where	Practice and instruction	Practice and instruction	Performance

## IMPLICATIONS FOR MUSICIANS: APPLYING CONCEPTS OF ERROR MANAGEMENT TO MUSICIANS

### A CONCEPTUAL RISK MANAGEMENT FRAMEWORK FOR MUSICIANS

The role of errors in music performance is comparable with the role of errors in higher risk disciplines such as aviation and medicine. Both cases refer to dynamic complex systems in which large amounts of data are quickly processed. Both involve psychological distress in response to errors or the threat of errors. Like errors in aviation or medicine, errors in musical performances (e.g., competitions) can have specific, irreversible consequences. Musicians have to deal with expected and unexpected incidents, and external and internal threats. Errors can have specific, severe, and irreversible consequences. Errors in music performance cannot be compared with the fatal errors that sometimes occur in hospitals. Errors in performance may nevertheless give a musician the feeling that their career is over and dead, and in extreme cases errors may indeed end a musical career. In this regard, even music performance may be seen as a high-risk discipline.

Balancing risks and managing errors may represent one of the core competences of musicians. But the latent underlying risks of performance situations are often ignored and underestimated, as the following anecdote illustrates. A violinist practiced very hard but nevertheless failed an audition. She attributed her failure to errors in her performance, and attributed those in turn to the following three events: shortly before the audition she met a hostile colleague; the accompanist played much faster than expected; and the committee was noisy, which was distracting. The errors could have been avoided if these variable factors had been anticipated along with appropriate response strategies, and if the corresponding skills had been practiced in prior simulations. It follows that musicians need specific training to achieve situational awareness and skills for coping with threatening situations. These skills and training could positively impact performance quality while at the same time reducing the associated psychophysiological costs. Proactive techniques attempt to detect, evaluate, and mitigate risk factors facilitating the avoidance of incidents (Kallus, 2012).

In risk management, disturbances are dealt with in advance to achieve better adaptability and flexibility, known as good error management. Applying the conceptual framework of risk management and an appropriate checklist prevents errors. That also reduces emotional strain and mitigates the consequences of errors by anticipating the identified risks.

The musician in the audition was surprised by the disturbances, provoking a *Rumpelstiltskin effect* (e.g., Fuchs, 2008). The latent threats and risks were overlooked. The musician failed to actively develop realistic expectations for the upcoming performance. The musician could have avoided the negative experience by pro-actively training more productive behavioral responses. We cannot predict the future, but we can train relaxed, flexible, creative responses to threats. Training in risk management would have given the performer skills in anticipatory processes. She would have taken risks consciously and purposefully with the aim of achieving or exceeding the goals of the music performance.

The educational researcher Smyth (2004) has argued that we need a conceptual framework to guide and structure our inquiries and to give orientation to collective ideas about a topic, e.g., error and risk management. A conceptual framework can help educational researchers to identify, clarify, and communicate questions and aims (Smyth, 2004). **Table 2** illustrates the main categories of risk management.

**Table 2:** A conceptual risk-management framework for musicians.

External threats	Stages of playing/ task-orientated categories	Internal threats
•Social factors	•Perceptual level	•Individual risks
•Material factors	•Kinematic level	•Psychological and physiological risks
	•Ergonomic level	
	•Acoustic level	

Three aspects of musical risk management are represented in this conceptual framework. First, there are external aspects with social and material factors. Social risks are mostly communication problems between musicians, students, and teachers; they involve missing or wrong information, or misinterpretation. Material factors can refer to problems with the instrument, the room, or the musical score. Task-oriented strategies are divided into different aspects of performance. Finally, there is the internal, person-oriented dimension, which includes individual risks and psycho-physiological aspects.

The next three sections address this conceptual framework in more detail and give practical implications for musicians. We will demonstrate a pragmatic form of risk management that considers a range of music performance risks in typical situations, as well as factors that musicians might take into account, when anticipating or simulating expected or unexpected risks, and practicing possible responses (see also Repp, 1996).

#### External threats

Possible external threats to a performance include social and organizational factors (e.g., accompanist does not play as expected; jury/audience distracts performer; performance space is too small; players in the ensemble have unclear intentions, e.g., whether to repeat a section; one player spontaneously provokes the other players into performing in a certain way; cultural differences in norms or standards are not clear). Material factors include trouble with the instrument (slippery fingerboard, broken string, poorly tuning, instrument breaks), problems with the room (poor or stifling air, poor lighting), and quality of musical score (misprint, poor layout).

Performers can use a list of this kind as a source of ideas for planning responses to different external constraints and performance situations. Accompanists and ensemble partners often play differently than expected. One should prepare for such situations by practicing at different tempos, dynamic levels, degrees and kinds of expression, and so on. Risks that could have major consequences but are relatively unlikely to happen are often “insurable risks” (e.g., when the instrument breaks or another musician cannot play in the performance). Risks that combine high likelihood with high consequences are “risks actively detecting, monitoring and mitigating” error consequences (Hirai, 2010). Musicians should practice recognizing and prioritizing significant risks and identifying the weakest critical controls.

#### Task-orientated categories in the stages of playing

The error classifications of Palmer and van de Sande (1993), Repp (1996), and Flossmann and Widmer (2011) mentioned in the first part of the review allow us to summarize task-orientated strategies in the different stages of playing music. Thus, musical performance can be broken down into a series of tasks or levels in which errors can occur (Repp, 1996). The authors list here some pragmatic examples for the four levels. First, we can distinguish between the perspective of visual perception and cognition (mainly score reading). Weak points are for example beginnings and ends of phrases, similar or identical passages, technically more difficult sections, and fortissimo passages. Second, we may consider the timing and position of movements and anticipation of movements (too late or too early) including the kinematics of arm and fingers including anticipation, perseveration, substitutions, omissions, and intrusions. Third, there are technical or ergonomic factors such as the force required producing a tone with a given loudness or fingering errors. Finally, we need to consider the acoustic level, which depends on the room. Musicians should practice performing in different room acoustics and they should be able to regulate the acoustic balance.

#### Internal threats

In the following, the authors list further individual predictors of risks. They are mostly caused by lack of proficiency, technical deficiencies, a destructive, negative attitude to errors, poor time management during practice or quick study. Most physiological challenges are cold or sweaty hands, trembling extremities or shortness of breath. Expectation depends on relative willingness to take risks, unexpected deviations between performance and expectations, unrealistically high expectations, negative outlook, and exaggerated beliefs in automation. Readers should feel free to revise this list in accordance with their own risk factors.

These are not the only things that can hinder a good performance. Additional factors include the failure to effectively manage fatigue and stress, negative attributions, performance anxiety, lack of concentration, attention deficit, and low self-efficacy. There are also extraordinary risks that happen under specific circumstances, as in an open-air concert in which a musician’s long hair may fall in front of her or his face when the wind blows. Musicians can prepare for such situations by practicing performing under similar circumstances and learning to anticipate such events. In this way, they can mitigate the negative consequences by developing behaviors that help them manage such incidents.

Some physiological symptoms of stage fright cannot be eliminated completely, but appropriate cognitive appraisal can minimize the consequences of such distracting indicators as trembling hands. Cognitive appraisal of a situation is a critical element in determining whether a performer regards an event or situation as stressful (Kenny, 2004). Performers need to confront the psychological aspects of different performance situations. The more that musicians and their teachers know about the causes of errors, and about the psychological correlations of expectation, thoughts and emotions, the more they can adjust their mind sets and reinforce their beliefs in a good performance.

### MANAGING ERRORS IN THE STAGES OF PRACTICE

Research in music psychology suggests the need to differentiate between different stages of practice (Ericsson et al., 1993). **Table 3** is a simple model of practice stages that focuses on dealing with errors. The model includes three stages that are error-friendly (or at least error-tolerant), and one stage (procedural learning) that is explicitly intolerant of errors.

**Table 3:** Stages to expertise in terms of handling errors (Kruse-Weber and Parncutt, 2013).

Stage of practice	Managing errors
*Exploration/Deliberate Play*	*No immediate correction of errors* Errors tolerated
*Declarative learning*	*Errors give information* Error friendliness
*Procedural learning*	*Acquisition of error management* **Errors avoided**
*Creative practice*	*Inventions and innovations* Errors friendliness

The first stage is one of *exploration* or *deliberate play*. Baker et al. (2003; cited in Hagemann et al., 2007) reported that experience in deliberate play in team ball sports at the beginning of a professional career in a different domain (e.g., sport) enhances career performance. Errors in this preliminary stage of deliberate practice are tolerated and not yet corrected. Research in music is consistent with these findings; children who later become professional musicians are primarily motivated by the joy of music (McPherson, 2001, 2007; McPherson and Davidson, 2006; Jabusch et al., 2007; Lamont, 2011).

*Declarative learning* is the acquisition of facts: knowledge about what, where, and when. In this stage, errors play an important role; teachers should give orientation and informative, constructive, concise feedback. Learning can be either explicit (declarative), in which the learner becomes aware of (and can describe) learning conditions and goals, or implicit (procedural), in which learning occurs below the learner’s level of awareness. *Procedural learning* is oriented toward error avoidance; skills and knowledge are acquired by repeated performance and practice.

The *creative stage* of practice is characterized by exploring new ideas, balances, tempos, sounds, and so on. “A practical implication of the current research is that educators and learners should introduce challenges into learning situations, including using tests as learning events, even if doing so increases initial error rates” (Kornell et al., 2009, p. 997). In the creative stage, risk-taking with challenging tasks can create new learning opportunities. At this stage, general principles of success in entrepreneurship can be applied to the musical environment to achieve greatness in performance. Creativity involves changing routines and paying attention to the moment during each performance. Psychologist Kallus (2012) has pointed out that psychomotor coordination must be largely error-free to enable the performer to handle errors in attention and anticipation; if we first achieve motor perfection, we are in a better position to predict errors.

### EXTENSION OF THE RANGE OF SOLUTIONS: NOVICES VS EXPERTS

**FIGURE 1:**
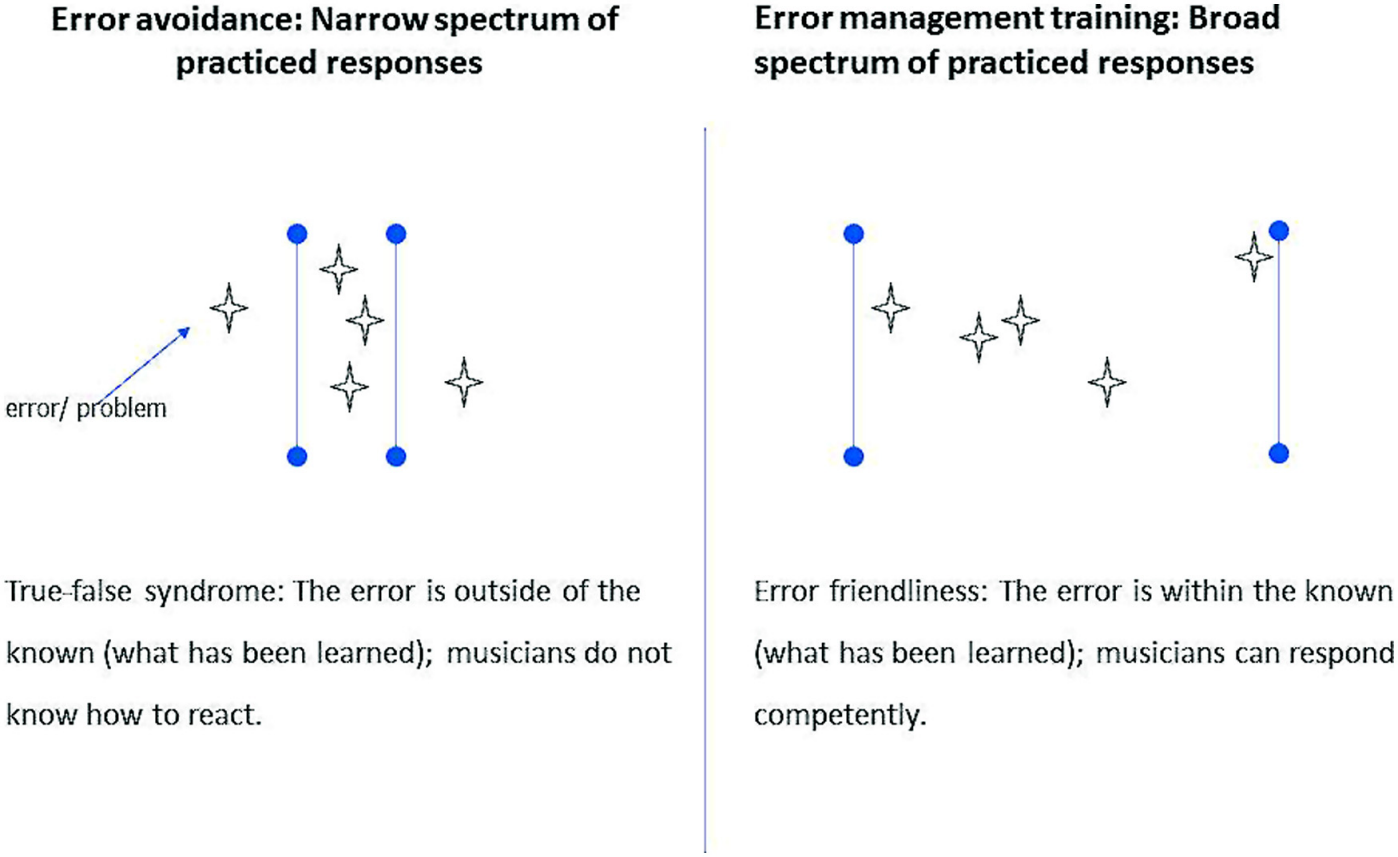
**Extension of the range of solutions (Kruse-Weber, 2012a)**.

Novices and experts approach errors differently. Novices may ignore errors and keep playing due to their lack of having appropriate schemata against which to evaluate progress (Pitts et al., 2000; Hallam, 2001; McPherson and Renwick, 2001; Barry and Hallam, 2002; in Hallam et al., 2012). Experts tend to creatively set goals, exploring and experimenting with musical parameters and techniques. They extend the range of solutions beyond its normal limits, monitor their progress and lapses in concentration and motivation in a self-supportive way, and finally develop a positive and relaxed attitude to errors taking challenging and creative tasks. By focusing on the most precise interpretation of the score, instead of additionally exploring sounds and colors, a performance might lose individual expression. An exaggerated focus on avoiding errors may lead to the “true-false syndrome” (Mantel, 2003, p. 56): If an error occurs that lies beyond the performer’s experience and expectations, the musician does not know how to react (**Figure 1**, left). Incorporation of systematic error management into the creative stage of deliberate practice can be a useful complementary strategy to prevent errors (**Figure 1**, right). The error lies within the anticipated range of possibilities. An extended number of solutions allow musicians to manage and fix errors faster and more easily. By promoting an anxiety-free approach to error analysis, a positive error climate is created.

In a holistic, non-linear, error-friendly approach, teachers should not break up a physical movement into sub-movements and have the student train them separately and put them together at the end. It is more productive for the learner to try out different solutions (e.g., different fingerings) him- or herself. As described in the differential learning approach of Schöllhorn et al. (2012), movement variations are the basis of learning rather than movement repetitions. They are realized by adding stochastic perturbations to a central movement pattern in order to avoid precise movement repetitions and corrections during the skill acquisition process. A differential approach is more beneficial because it steers learners toward more functional movement patterns during practice (Schöllhorn et al., 2012).

The most important strategy for performance is to “practice performing!” (Norris, 2009, p. 24). Practicing performing and developing a good error management needs extension of the usual range of solutions and creative or challenging physical or mental coordination exercises (Westney, 2003). A list of examples include playing with closed eyes, mental training, performing music after sport (with high blood pressure), performing in the middle of the night, performing with errors and blackouts, and performing while standing on one leg (e.g., Norris, 2009). The most advanced stages of practice leading to performance should take on challenging tasks in an error-positive climate. Different approaches can be combined: improvisation, invention, exploration, and risk-taking. Garnett (2013) describes this in Jazz improvisation: “Yes, risk invites actions that others might later say are “mistakes”. But success requires taking smart risks. That means being courageous enough to take risks when you should. AND it means being courageous enough to step away from risks you shouldn’t take. (…) Improvisation focuses on being willing to be in the moment and respond to what’s going on around you. These risks deliver a great product to listeners.”

### ERROR MANAGEMENT TRAINING AS METACOGNITION IN MUSIC EDUCATION

In risk and error management from other disciplines such as aviation, errors are accepted as humans. Errors are expected, tolerated, and regarded as informative for the learning process; error management is incorporated into the training procedures. Similarly, musicians could benefit by developing cognitive monitoring skills and reflecting upon their thoughts during practice, their opinions about errors and attitudes toward skill acquisition.

Keith and Frese (2008) showed that EMT is more effective in well-structured tasks with clear feedback opportunities. This suggests that the discussion and analysis of errors should play an important role in instrumental teaching. The learner should be encouraged to think independently about dealing with errors. Guidelines and questions should be brief to give the learner space to develop individual metacognitive skills. Music teachers should encourage students, through a structured lesson format, to adopt a self-reliant attitude to a reflective practice and metacognition skills (Barry and Hallam, 2002; Kruse-Weber, 2012b). Thus, EMT can help musicians practicing at home without an instructor. They learn to reflect on errors and to develop monitoring their skills on their own. Instead of merely avoiding errors, they regularly determine the strengths and weaknesses of their performances to find out what improvements are needed for the next session. They must be able to accurately evaluate their own performance (Burt and Mills, 2006; in Hewitt, 2009).

Flavell (1979) describes strategies for promoting metacognition such as self-questioning (e.g., what do I already know? how have I solved problems like this before?) and thinking aloud while practicing. The physical act of exploring and playing could play a large part in the development of metacognitive skills (Carr, 2002; in Flavell, 1979). According to this approach, music educators should emphasize the non-technical, invisible skills of procedural verbalization and self-reliance in strategic decision making through a structured lesson format. They should provide metacognitive experiences especially in stimulating conscious thinking situations.

According to Flavell (1979), declarative metacognition involves knowledge about the person, the task, and the strategy. Person-related knowledge refers to everything one knows about his own thinking and memory and task-related knowledge. The executive aspect of metacognition involves the metacognitive control (self-regulation) and the control (self-monitoring). The control aspect determines whether one is on the way to target in terms of planning milestones that approach the final goal (Flavell, 1979). Recent research in music education and music psychology has suggested that self-regulation should be taught from the beginning of learning a musical instrument by, e.g., teachers and parents and that it is a key for musical success (McPherson, 2012).

Developing a culture of error-management training in instrumental music education can open up explicit perspectives of metacognition, e.g., talking about errors, sharing knowledge about errors, sharing strategies to manage errors, shared support, quick error detection and analysis, effective recovery from errors, and coordinated handling of errors (Van Dyck et al., 2005). Thus, metacognition in music performance can be seen as an important ingredient in the development of complex performance skills. It is an ongoing process of planning, checking, monitoring, attending to, revising, and evaluating thought processes so as to optimize them and minimize errors, and respond to errors more quickly, creatively, constructively, and effectively.

For instance, children might at first distinguish only between understanding and not understanding things; they might know only being confused, unable to act, uncertain about what is intended or fail to discern what they should do next (Flavell, 1979). Experiences from the researchers with instrumental music lessons assume that young children from 6 to 8 already have a quite high potential to develop their knowledge, comprehension, and self- regulation about cognitive phenomena specifically in sight-reading. Children show that they can establish new goals and revise or abandon old ones. While communicating about metacognitive experiences the young learners can add, delete, or revise their strategy. Instrumental music teachers who are already specifically (intuitively) doing what we are recommending interact and communicate about the thoughts and attitudes from the student in the processes of problem solving.

Error management training is an additional means of promoting metacognitive skills in the area of self-regulation. Musicians can learn through EMT to better manage the negative consequences of their errors. The benefits include more security and more effective decision making.

## CONCLUSION

Students and musicians can be made more aware of the informative nature of their errors. They can realize for example that a piano keystroke which sounds wrong (whether in terms of pitch, timing, or intensity) has a complex cause that they can analyze in order to systematically prevent similar errors in the future. Empirical error studies in music performance have allowed errors to be classified into categories (Palmer and van de Sande, 1993; Repp, 1996; Flossmann and Widmer, 2011), yielding insights into the planning and execution of complex tasks as, e.g., (piano) performance. The differentiations of these categories imply that a lot can be learned from their analysis in specific practical situations. Maidhof (2013) investigated how musicians monitor their errors – how they detect deviations from intended sounds. He showed that errors can be immediately perceived – even before the result of an erroneous action manifest itself. Thus, auditory feedback is not a prerequisite for the detection of errors made during music (piano) performances. We assume that metacognition and cognitive monitoring are the further key aspects of error detection.

Further research is needed to describe and explain spontaneous developmental acquisitions from learners and find effective ways of teaching metacognitive knowledge and cognitive monitoring skills in instrumental music pedagogy. Finally, the competency specifications should be empirically validated and then again compared with the investigations in other forms of professional training (e.g., aviation) to test whether there really are benefits and clarify the differences between approaches of different disciplines. On this basis, we would be able to develop realistic strategies to improve instrumental pedagogy.

We should focus on the process of learning to manage errors rather than merely cataloging metacognitive inadequacies (see also Flavell, 1979). Error management in non-musical fields can clarify aspects of musical error management and open up new perspectives for further research in music education and music performance. Non-musical research and applications suggest that risk and threat management generally improves performance. Error management training promotes skill transfer from one task to another, because errors during training stimulate attention, which in turn facilitates later retrieval of similar problems and their solutions.

We have summarized error management concepts in different disciplines. Then we outlined three new lines of research since the 1990s in organizational psychology: risk management (before the error), error management (after the error), and EMT (during and after the error). We then tailored these approaches to the needs of musicians and attempted to bring together psychological and pedagogical approaches and practical concerns about these error management concepts. By so doing, we have added a new dimension to existing approaches to error in music performance and music education, opening up and rethinking unilateral negative attitudes toward errors in teaching and traditional (behavioristic and social cognitive) learning theories. We have argued that constructive, differential learning approaches are appropriate terrain for the development of metacognition.

We have set out a conceptual framework for musical risk management that gives orientation to musicians by identifying, clarifying, and communicating error issues. Our concept separately addresses internal and external threats and risks, and task-orientated strategies in different stages of practice. Detailed practical examples for developing a repertoire of responses to latent risks can allow a musician to deal effectively with performance threats when they arise. A list of possible individual risks should be generated and crosschecked by the student while rehearsing appropriate, sensitive responses to the anticipated incidents. Systematic risk management training can improve attention and awareness for performance traps and knowledge of risks that apply only to certain kinds of music or performance, or even to an individual performer. Realistic expectations about errors can prevent errors from occurring; awareness of anticipatory processes can reduce emotional stress. Following this kind of preparation, musicians are more able to take risks during performances and respond spontaneously to the situation in which they find themselves while performing. That can help them improve their technique, expression, and interpretation.

Error prevention is more than mere errorless learning and error avoidance in performance. It is a complex process that paradoxically involves error tolerance. We need to examine the paradox that EMT can provide fundamental skills for error prevention. Atkinson (1957) argued that performances can be “greatest when there is greatest uncertainty about outcome”: people with a strong motivation to achieve prefer immediate risk, whereas those with strong motivation to avoid failure prefer easy tasks or extremely difficult and risky tasks.

The learning and teaching approaches of EMT are relevant for both novices and experts in music performance. Music academies should offer workshops for top-level performers, to better evaluate their performances by evaluating and developing metacognition and monitoring skills. Students in such workshops would be active participants. With the support of researchers and teachers, they would creatively and collectively generate lists of difficult situations that lead to errors in their performance. They would consider the skills that they need to respond creatively to such situations as they occur, and how these skills could be acquired by practice.

Research in other disciplines confirms that exploration in combination with metacognitive and task-orientated strategies, positive experiences of failure, and error-friendly working conditions can promote successful learning and performing. Teachers should not only tolerate errors – they should encourage students to make errors in an atmosphere of trust and mutual respect. Metacognitive skills may be best developed by a combination of low blame, no punishment, and a high degree of empathy in the classroom setting. In instrumental music instruction, we should promote analysis and discussion of errors during teaching, so as to utilize them strategically as potential learning opportunities.

It is understandable that music academies focus on noticeable effects such as errors and their avoidance and neglect the invisible processes of metacognition. Research suggests we should be paying more attention to the contents of the psychological black box. Students need more support in developing their skills of cognitive monitoring to enable them to implement everyday instructions to relax, pay attention to the musical structure, read the score correctly, use the correct fingering, and so on.

This contribution has aimed to develop a new understanding of errors and the error process in music performance and instrumental music education from which performers, teachers, and students can benefit. Our findings highlight the importance of developing a systematic approach to error management so as to rationally deal with errors and to learn from them. With appropriate teacher training, the ideas can be incorporated into instrumental music teaching. It is feasible to increase the amount of teaching time that is devoted to the development of metacognitive knowledge and monitoring skills without increasing the total teaching time and achieve a better overall result. Adaptation to these principles could be made gradually in a process of evolving optimization. The development of metacognitive skills including error management can feasibly be listed among the main goals of music academies.

## ACKNOWLEDGMENT

This work was financially supported by University of Music and Performing Arts Graz, Austria.

## Conflict of Interest Statement

The authors declare that the research was conducted in the absence of any commercial or financial relationships that could be construed as a potential conflict of interest.
